# The mid-childhood and adolescent antecedents of women’s external locus of control orientation

**DOI:** 10.12688/wellcomeopenres.12052.2

**Published:** 2017-11-06

**Authors:** Jean Golding, Steven Gregory, Yasmin Iles-Caven, Stephen Nowicki

**Affiliations:** 1 Centre for Child and Adolescent Health, School of Social and Community Medicine, University of Bristol, Bristol, UK; 2 Department of Psychology, Emory University, Atlanta, GA, USA

**Keywords:** ALSPAC, exposome, locus of control, life events, parental smoking, infancy, childhood, adolescence

## Abstract

**Background**: External locus of control orientation (ELOC) is a powerful predictor of adverse consequences in regard to health, educational attainment, inter-personal relationships and well-being. Although many cross-sectional studies have been carried out, relatively little is known about antecedent factors influencing the development of ELOC.

**Methods**: Over 12,000 pregnant women who enrolled in the Avon Longitudinal Study of Parents and Children (ALSPAC) in south-west England, had completed a brief version of the Adult Nowicki-Strickland Internal-External LOC scale, together with detailed questions concerning their own parents and childhood.  A series of hypothesis-free structured backwards stepwise logistic regression analyses used an exposome approach with ELOC as the outcome.

**Results**: Significant positive associations were found with smoking of the parents of the surveyed women, including prenatal exposure, and their own onset of regular smoking in mid-childhood (6-11 years). Increased odds of ELOC were also found with the absence of their fathers in early childhood, presence of older siblings, and with being born and brought up in the same area as they resided in at the time surveyed. Protective influences in the surveyed women included positive rating of their mother’s care, having a relatively educated mother, attending boarding school, their own age (the older they were, the less likely were they to have an external orientation), having a mentally ill parent, a sibling hospitalized or a relative die.

**Conclusions**: There are two conclusions: (i) that not all stressful events contribute to the development of ELOC and it would be essential for models of antecedents of ELOC to take note of this complexity, and (ii) there are consistent (albeit unexpected) findings that highlight associations with cigarette smoke exposure of the woman from fetal life through to when starting to smoke regularly herself in mid-childhood. It is important that these findings are tested in other populations.

## Introduction

Locus of control (LOC) refers to individuals’ generalized expectancy regarding the connection between their behavior and its consequences in a problem solving context. Those who fail to see a connection between what they do and what happens to them, and instead view what happens to them as the result of luck, fate, chance, or powerful others are seen as externally controlled (ELOC). Conversely, those who tend to perceive a connection between their efforts and what happens are called internally controlled (ILOC).

Over the past 50 years since its introduction, LOC has proven to be one of the most popular topics for researchers who have found it to be significantly related to an ever growing number of important and significant aspects of human life, including personality characteristics, social adjustment, academic achievement, health, and business success ( Lefcourt, 1982; Lefcourt, 1983; Nowicki, 2016a; Nowicki & Duke, 2016; Rotter, 1966; Rotter, 1975; Rotter, 1990). Because of its extensive association with important outcomes, it would be helpful to identify and understand some of the possible antecedents of locus of control.

The present study sought to respond to the identified need for a study of LOC antecedents within a larger diverse population by using data from over 12,000 pregnant women enrolled in the Avon Longitudinal Study of Parents and Children (ALSPAC) ( Boyd *et al.*, 2013; Golding *et al.*, 2001). In the present study, we are defining locus of control of reinforcement as the cognate introduced by Rotter (1966). ALSPAC is a population based study which has followed parents and their offspring for over 20 years, collecting a wealth of information concerning environmental exposures, both physical and psychological, and a variety of phenotypes including measures of locus of control. The cohort includes families of differing social backgrounds and beliefs, and is representative of the residents of the local area, encompassing urban and rural communities, rich and poor, young and old parents.

In our initial attempt at using this dataset to identify possible antecedent factors of ELOC in adult women, we highlighted a number of features of their parents and early childhood. ( Golding *et al.*, 2017). The associations went beyond the usual personality/child rearing attitude indicators identified in previous studies, and found external LOC to be related to: the years in which the women’s parents were born (the more recent, the more likely they were to be externally oriented); the education levels reached by each of their parents (those whose parents were less educated were more externally oriented); whether either of their parents was a smoker, and in particular whether their mothers smoked prenatally; the womens’ own year of birth; the number of older siblings the mothers had; and whether their father was absent from home in the first years of their childhood.

In the present study, we continue our search to clarify antecedents of LOC by focusing on events, familial behaviors and other features of *later* childhood and adolescence, particularly stressful environments and traumatic events, that may be associated with the locus of control of the adult women on whom we reported in the earlier study ( Golding *et al.*, 2017). We also evaluate whether characteristics from early childhood predictive of adult ELOC sustain their impact at a later age, or whether they are themselves predictive of factors that have a more direct effect on the development of ELOC.

Our hypotheses were that:

(i) The factors described by Carton and Nowicki in 1994 (of low levels of parental control, low levels of stress, especially involving father absence, warm parenting and parents who rewarded and punished consistently and contingently) would be found to be negatively associated with the ELOC of the women;
(ii) These factors would ‘explain’ the associations found in regard to their early childhood and the parental background; and
(iii) No other environmental (physical or psychological) exposure considered would be independently associated with ELOC.

## Material and methods

### The ALSPAC study

This pre-birth cohort was designed to determine the environmental and genetic factors that are associated with health and development of the study offspring ( Boyd *et al.*, 2013; Golding *et al.*, 2001). Pregnant women with an expected date of delivery between April 1991 and December 1992 and residing in Avon (UK) were invited to take part. Because it was thought that features of the birth of the baby, and any difficulties involved, might alter the parents’ responses in regard to their attitudes and behaviors, there was a concerted effort before the child’s birth to obtain details of their personalities, moods and attitudes, including a measure of their LOC. Data were collected throughout the longitudinal study at various time-points using self-completion questionnaires, biological samples, hands-on measurements, and linkage to other data sets. For full details of all the data collected see the study website: www.bristol.ac.uk/alspac/researchers/data-access/data-dictionary/.

### Ethical approval

Ethical approval for the study was obtained from the ALSPAC Ethics and Law Committee (unnumbered) and the Local Research Ethics Committees: Bristol and Weston Health Authority: E1808 (28 ^th^ November 1989); Southmead Health Authority: 49/89 (5th April 1990) and Frenchay Health Authority: 90/8 (28th June 1990).

### The outcome measure

The LOC measure used in the present study is a shortened version of the adult version of the Nowicki-Strickland Internal-External locus of control scale (ANSIE). This comprises 40 items in a yes/no format, which assess perceived control ( Nowicki & Duke, 1974). It was chosen over other scales more specifically related to perceived control over health, as it was considered that this more generalized scale would relate to other factors in addition to health outcomes. Construct validity for the scale has been found in the results of over a thousand studies ( Nowicki, 2016b). The version used in the present study comprises 12 of the original 40 items, selected after factor analysis of the ANSIE administered as a pilot to 135 mothers in the USA. This was completed by women during their pregnancies. From the responses LOC scores were calculated, the higher the score the more external the locus of control; conversely the lower the score, the more internal her orientation. The scores ranged from 0 to 12. The frequency of responses for the women was roughly normally distributed with a median of 4. For this study external locus of control was defined as having a score greater than the median. This cut-off identified 45.2% of the women as externally controlled (ELOC).

### The antecedent variables considered

In this paper, we considered four different groups of variables pertaining to:

(a) the demographic background of the women;

(b) their mid-childhood (6 – 11 years);

(c) their adolescence (12 – 16); and

(d) traumatic events that occurred at any stage during their childhood (<17 years).

The details of the variables considered are described in Supplementary File 1.

### Statistical analyses

The following analyses were undertaken sequentially:

(i) The unadjusted associations with ELOC were calculated for each group of variables;
(ii) The variables with unadjusted p-value <0.05 were selected and offered to a backward logistic regression for each group;
(iii) The results for each group were considered in regard to the numbers of individuals left in each regression and variables were either dropped or recoded to increase the numbers available in the regression where feasible;

Once these intra-domain regressions were finalized, the groups were combined for inter-group analyses in a similar way to our earlier publications (e.g. Golding *et al.*, 2014). Comparison of goodness-of-fit (GOF) between the analyses used 100 times the pseudo-R ^2^ statistic: the higher the value, the better the fit.

## Results

### Timed features of childhood

In our earlier paper we showed that there were aspects of early childhood (birth to 5 years) that were independently associated with the women’s ELOC ( Golding *et al.*, 2017). These comprised year of birth, whether they were born in Avon, breast fed (protective), had a birthmark, had at least two older siblings and whether their father was present in the household ( Supplementary Table 1). The model had a GOF of 5.79.

Here, we examine the features of two later stages of childhood – from 6 to 11 years, and from 12 to 15 years. The unadjusted data that show statistically significant relationships are shown in Table 1; they demonstrate strong associations with separation or divorce, presence of either biological parent, a step-father, step-sibling, mother’s partner, whether they had started smoking regularly during the period, whether menarche had occurred, and how happy or unhappy they may have been.

**Table 1.** Unadjusted associations between proportions of women with ELOC and features of mid-childhood (6–11 years) and adolescence (12–15 years).

Childhood experiences	Mid-childhood (6–11 years)			Adolescence (12–15 years)		
	%(n) ELOC	OR [95% CI]	P	%(n) ELOC	OR [95% CI	P
*Mother Present in Home*	N=12638		<0.0001	N=12638		<0.0001
Yes	43.8% (5122)	0.45 [0.39, 0.52]		44.1% (5173)	0.52 [0.45, 0.59]	
No	63.3% (596)	1.00 Ref		60.4% (545)	1.00 Ref	
*Father Present in Home*	N=12638		<0.0001	N=12638		<0.0001
Yes	43.0% (4657)	0.52 [0.47, 0.58]		42.9% (4412)	0.60 [0.55, 0.66]	
No	58.9% (1061)	1.00 Ref		55.5% (1306)	1.00 Ref	
*Step-father Present in Home*	N=12638		<0.0001	N=12638		<0.0001
Yes	54.8% (269)	1.49 [1.24, 1.79]		52.9% (397)	1.38 [1.19, 1.60]	
No	44.9% (5449)	1.00 Ref		44.8% (5321)	1.00 Ref	
*Step-brother Present in Home*	N=12638		<0.0001	N=12638		<0.001
Yes	60.6% (109)	1.87 [1.39, 2.53]		56.2% (141)	1.57 [1.22, 2.01]	
No	45.0% (5609)	1.00 Ref		45.0% (5577)	1.00 Ref	
*Step-sister Present in Home*	N=12638		<0.0001	N=12638		<0.001
Yes	61.7% (87)	1.96 [1.40, 2.76]		58.3% (126)	1.71 [1.30, 2.18]	
No	45.1% (5631)	1.00 Ref		45.0% (5592)	1.00 Ref	
*Mother’s Partner Present*	N=12638		<0.0001	N=12638		<0.0001
Yes	61.4% (94)	1.94 [1.40, 2.70]		57.9% (139)	1.68 [1.30, 2.18]	
No	45.0% (5624)	1.00 Ref		45.0% (5624)	1.00 Ref	
*Parents Divorced/Separated*	N=12424		<0.0001	N=12424		<0.001
Yes	55.3% (430)	1.54 [1.33, 1.78]		52.5% (270)	1.36 [1.14, 1.62]	
No	44.6% (5192)	1.00 Ref		44.9% (5352)	1.00 Ref	
*Started smoking regularly*	N=12184		<0.0001	N=12184		<0.0001
Yes	74.6% (121)	3.59 [2.53, 5.12]		63.5% (1224)	2.46 [2.23, 2.72]	
No	44.4% (5348)	1.00 Ref		41.4% (4245)	1.00 Ref	
*Menarche*	N=12635		0.006	N=12635		<0.0001
Yes	47.8% (1081)	1.14 [1.04, 1.24]		43.9% (3731)	0.85 [0.79, 0.91]	
No	44.7% (4636)	1.00 Ref		48.0% (1986)	1.00 Ref	
*Recollection of happiness*	N=12529		<0.0001	N=12579		<0.0001
Very happy	41.6% (3351)	1.00 Ref		42.0% (2328)	1.00 Ref	
Moderately happy	49.4% (1164)	1.37 [1.27, 1.49]		45.1% (2100)	1.14 [1.05, 1.23]	
Not really happy	58.6% (412)	1.99 [1.70, 2.33]		52.6% (719)	1.54 [1.36, 1.73]	
Unhappy	60.3% (237)	1.95 [1.55, 2.36]		54.9% (549)	1.68 [1.41, 2.03]	

Mutual adjustment within each age group resulted in small numbers of variables remaining in the models ( Supplementary Table 2 and Supplementary Table 3). For mid-childhood, these comprised presence of mother in the household (OR 0.59, 95% CI 0.50-0.70), father in household (OR 0.71, 95% CI 0.63-0.81), unhappiness in childhood (OR 1.27, 95% CI 1.20-1.33) and smoking regularly by age 11 (OR 3.16, 95% CI 2.19-4.58). Together with a small association with menarche by age 11 (OR 1.11, 95% CI 1.01-1.22), this model had a GOF of 1.99. Similarly, during adolescence independent associations concerned the absence of the father (OR 1.44, 95% CI 1.31-1.59) and/or of the mother (OR1.62, 95% CI 1.40-1.88), as well as level of unhappiness (OR 1.12, 95% CI 1.09-1.16); the GOF for this model was 1.26.

Combining variables from these two groups with those identified in the first 5 years of life ( Golding *et al.*, 2017) revealed 10 as independently associated ( Table 2). These indicated that the age at which the absence of the father was most predictive of ELOC was in early childhood, whereas absence of the mother was most relevant in adolescence. Their retrospective ratings of how happy they were indicated both mid-childhood and adolescence to be important. However it should be noted that the majority of associations were present in infancy or early childhood. This is illustrated by the data on GOF. These equaled 5.79, 1.99 and 1.26 for the three age groups respectively; the combined model had a GOF of 6.14, thus indicating that influences after 5 years of age made only marginal contributions to the overall model.

**Table 2.** Model predicting women’s ELOC after offering all variables in each of the three age-group analyses to a backward logistic regression. N = 8945, goodness-of-fit = 6.14.

VARIABLE	OR [95% CI]	P
*In early childhood*		
Year of birth	1.62 [1.51, 1.74]	<0.0001
Born in Avon	1.87 [1.70, 2.04]	<0.0001
Breast fed	0.86 [0.78, 0.94]	<0.001
Has birthmark	1.16 [1.05, 1.28]	0.004
No. older siblings	1.26 [1.13, 1.40]	<0.0001
Father absent	1.68 [1.39, 2.03]	0.009
*In mid-childhood*		
Smoked regularly <11 years	1.81 [1.16, 2.83]	0.009
Happiness	0.84 [0.78, 0.91]	<0.0001
*In adolescence*		
Mother absent	1.34 [1.08, 1.66]	0.008
Happiness	0.91 [0.86, 0.97]	0.002

### Relationship with further social features of childhood

A variety of features of childhood were ascertained for the study individuals (but without a measure of timing other than that they occurred before the age of 17). These included: whether they had attended a “special school” or boarding school; had been “in care”; length of time spent in hospital; were treated by a child psychiatrist, physiotherapist, or speech therapist; whether they had lived with grandparents, other relatives, friends, foster parents; resided in a children’s home, in custody or elsewhere; whether they left home before the age of 18; and unpredictability of their parents’ behavior. A set of 25 questions were used to assess their relationship with their mother using the Parental Bonding Index ( Parker *et al.*, 1979), from which two scores were derived – the maternal care score and an over-protectiveness score.

Among the women studied, there were increased risks of ELOC if they had attended a special school; been seen by a child psychiatrist; had speech therapy; had been in care; lived with grandparents or other relatives, friends or foster parents; were in a children’s home; had left home before age 18; or had an unpredictable mother or father. Conversely, they were much less likely to have an ELOC if they had seen a physiotherapist; went to a boarding school; stayed in an ‘other place’; or reported a positive maternal care score ( Supplementary Table 4). An intra-domain analysis resulted in 11 variables remaining in the model predicting ELOC including speech therapy; living with other relatives; living with foster parents; staying in a children’s home; having an unstable mother; and the home being unstable. The GOF was 2.84 ( Supplementary Table 5).

### Relationship with traumatic events

The ALSPAC study developed a set of childhood life events. This comprised a set of 31 specific items designed in a similar way to the life events inventory based on the earlier work of Coddington (1972). However for this study we omitted from the analyses the life events that may have been the result of having a high ELOC, including being pregnant, suspended from school and being in trouble with the police. There were 18 unadjusted associations ( Supplementary Table 6), eight of which were eliminated on intra-domain analysis. The remaining significant positive factors were: death of a friend; parent had a serious accident; being physically abused by a parent; divorce of parents; and being sexually abused. Apparently protective features were: a parent was mentally ill; moving to a new district; death of relative; and hospitalization of a parent or a sibling ( Supplementary Table 7). The GOF was 1.94.

### Combination of social environment, traumatic events and timed childhood features

Offering a combination of the 11 social, 10 traumatic and 10 timed childhood data to a stepwise logistic model revealed 18 variables that remained associated. Of those that failed to enter were the mother and father being absent from the household at different time points; menarche by 11; happiness in adolescence; being sexually abused; moving to a new district; attending a special school; seeing a physiotherapist; being in care; living with friends and having an unpredictable/unstable father ( Supplementary Table 8). The subsequent model is shown in Table 3. The 22 significant independent associations included 16 that had a p-value <0.01. These comprised positive associations with: the women’s year of birth (which is equivalent to the woman’s age since the measures of LOC were all made between 1991 and 1992); whether they had been born in the study area (Avon); the number of older siblings; whether they had a birthmark; whether their father was absent from the household in early childhood; whether they lived with grandparents; left home before the age of 18; had a friend die or a parent had a serious accident. In contrast, apparently protective features at p < 0.01 were having been breast fed; having a happy mid-childhood; going to boarding school; having a caring mother; death of a relative; hospitalization of a parent or sibling; and a parent being mentally ill. The GOF was 7.50.

**Table 3.** Model predicting ELOC after offering all variables relating to the women’s childhood using backwards logistic regression. N=8673; Goodness-of-fit = 7.50.

VARIABLE	OR [95% CI]	P
*From early childhood*		
Year of birth	1.53 [1.43, 1.65]	<0.0001
Born in Avon	1.81 [1.65, 1.99]	<0.0001
Has birthmark	1.19 [1.07, 1.32]	0.001
No. older siblings	1.26 [1.12, 1.41]	<0.0001
Was breast fed	0.87 [0.79, 0.96]	0.003
Father absent from household	1.61 [1.32, 1.97]	<0.0001
*In mid-childhood*		
Degree of happiness	0.88 [0.81, 0.95]	0.001
Smoked regularly	1.72 [1.06, 2.78]	0.027
*In adolescence*		
Mother absent from household	1.26 [1.00, 1.58]	0.046
*Social care*		
Attended child psychiatrist	1.36 [1.06, 1.76]	0.017
Lived with grandparents	1.40 [1.11, 1.76]	0.004
Went to boarding school	0.46 [0.35, 0.61]	<0.0001
Left home before age 18	1.41 [1.24, 1.59]	<0.0001
Stayed elsewhere	0.78 [0.64, 0.95]	0.016
Maternal care score	0.77 [0.72, 0.84]	<0.0001
*Traumatic life events*		
Relative died	0.85 [0.77, 0.93]	<0.001
Friend died	1.21 [1.07, 1.39]	0.004
Parent in hospital	0.90 [0.82, 0.99]	0.038
Sibling in hospital	0.84 [0.75, 0.94]	0.003
Parent had serious accident	1.45 [1.16, 1.82]	0.001
Was physically abused by a parent	0.73 [0.55, 0.98]	0.034
A parent was mentally ill	0.65 [0.51, 0.82]	<0.001

### Adding features of the women’s parents

In our earlier paper, we showed that some of the basic features of the women’s parents were major contributors as to whether they developed an external orientation or not. The independent features comprised the years of birth of each parent; their education levels; their ages at the study woman’s birth; the social classification of their father’s occupation; whether their father smoked; and whether their mother smoked prenatally ( Supplementary Table 9).

Adding these variables to the step-wise model which included the childhood variables meant that just 20 variables were retained in the model ( Table 4 and Supplementary Table 10). These included the following variables, associated with increased levels of ELOC at P < 0.01: their mother’s year of birth; their mother smoking prenatally; their father smoking, their father’s lower social classification; the women’s own year of birth; whether they were born in Avon; had lived with their grandparents; had left home before age 18 and whether they had experienced the death of a friend. Conversely, protective factors at P < 0.01 included the education level of their mother; whether they went to boarding school; the degree of maternal care the mothers felt had they received; whether a relative died; a sibling had been in hospital or a parent was mentally ill.

**Table 4.** Final model predicting ELOC including characteristics of their parents with those of their childhood. N=7285; Goodness-of-fit = 8.37.

VARIABLE	OR [95% CI]	P
*Features of her parents*		
Mother’s age <25 at birth	1.14 [1.07, 1.21]	<0.001
Mother’s education ≥O-level	0.66 [0.59, 0.75]	<0.0001
Mother smoked in pregnancy	1.17 [1.05, 1.30]	0.005
Father was a smoker	1.23 [1.09, 1.39]	<0.001
Father’s social group	1.08 [1.05, 1.11]	<0.0001
*In early childhood*		
Year of birth	1.38 [1.25, 1.52]	<0.0001
Born in Avon	1.61 [1.44, 1.79]	<0.0001
No. older siblings	1.19 [1.04, 1.35]	0.011
Father absent from household	1.35 [1.05, 1.74]	0.019
*In mid-childhood*		
Degree of happiness	0.90 [0.83, 0.98]	0.018
Smoked regularly	1.78 [1.01, 3.12]	0.045
*Social care*		
Taken into care	2.19 [1.19, 4.03]	0.011
Lived with grandparents	1.45 [1.12, 1.89]	0.005
Went to boarding school	0.57 [0.42, 0.78]	<0.001
Left home before age 18	1.32 [1.15, 1.52]	<0.0001
Maternal care score	0.79 [0.72, 0.86]	<0.0001
*Traumatic life events*		
Relative died	0.86 [0.77, 0.95]	0.003
Friend died	1.60 [1.25, 2.06]	<0.001
Sibling in hospital	0.84 [0.74, 0.95]	<0.001
A parent was mentally ill	0.64 [0.48, 0.84]	0.001

## Discussion

We have used a locus of control score conceptualized as a continuum from internality to externality and not as a typology of internals and externals. We have shown, using a sequential approach, that 20 descriptors of childhood were independently associated with ELOC as defined using scores greater than the median. The analyses used six different mutually distinct groupings, those providing the greater GOF were features of the parents and of early childhood ( Table 5). We have shown elsewhere that a combination of these two groups of variables increased the GOF to 6.89 ( Golding *et al.*, 2017); in Table 5 we show that each addition of the various measures increased the GOF, implying that all facets had a role to play in determining ELOC.

**Table 5.** Pattern of goodness of fit (GOF) measures within the different models (the higher the GOF, the better the fit).

Model	GOF	No. variables
Early childhood (EC)	5.79	6
Mid-childhood (MC)	1.99	5
Adolescence (A)	1.26	3
Social care (SC)	2.84	11
Life events (LE)	1.94	10
Features of parents (P)	5.74	9
EC + MC + A	6.14	10
EC + MC + A + SC + LE	7.50	22
P + EC + MC + A + SC + LE	8.37	20

### Timing of exposures in childhood

We have been able to identify the time when women were most likely to be influenced by circumstances of their childhood, such as the presence of their parents at home, and their recollection of unhappiness. Results are mixed, with absence of the father being most important in early childhood, and their degree of happiness in mid-childhood ( Table 4). In contrast, the events known to have occurred in adolescence did not appear in the final model. Unfortunately, we had no information on the timing of the traumatic events that occurred, which we had shown appeared to influence the development of ELOC.

### Smoking as a marker of risk

The data were consistent in indicating that the women who were externally oriented had an increased risk of (a) having a father who smoked; (b) having been exposed in utero to their mother smoking, and (c) being a regular smoker by the age of 11. These factors were all independently associated and were not explained by social conditions. This raises the question as to whether parents who smoke are themselves more externally oriented, and hence more likely not to try to stop their daughter from smoking in mid-childhood, or whether exposure of the girl to cigarette smoke had a biological effect on the developing brain resulting in susceptibility to ELOC. While it is most likely that there are psychological (e.g. modelling) and sociological (e.g. social class) reasons for parental smoking to be associated with ELOC, there is some reason to suggest a biological effect theory. Brain imaging techniques have shown that chronic tobacco smoking is associated with cortical volume, brain density and chemistry, and areas that involve executive function and memory ( Domino, 2008). A reduction in grey matter volume and density is reported among smokers ( Dome *et al.*, 2010). However, whether this explains why there may be a link between early exposure to cigarette smoke and ELOC is unclear, since to our knowledge, there have been no studies linking brain volumetric measurements to ELOC. The only report of any relevance to this is a study of 16 young adults who had been tested with a scale that combined self-esteem with internal LOC: the authors reported a significant correlation between this measure and hippocampal volume ( Pruessner *et al.*, 2005).

### Warmth of relationships with parents during childhood

Previous evidence for the importance of parental warmth, control, consistency and life stress in determining LOC (see Carton & Nowicki, 1994) was tempered by the fact that nearly all researchers obtained their data with self-report, usually retrospective cross-sectional methodologies and small homogeneous samples. Here, we use a much larger dataset than ever used before to address the question concerning the possible antecedents of having an externally oriented locus of control (ELOC), using a population of pregnant women in the UK, albeit still using self-report retrospective recall. The study results emphasize the importance of a woman’s relationship with her parents early in childhood. As predicted, we have shown that absence of the father from the household in the early years, being taken into care and living with grandparents during childhood were associated with increased risk of ELOC. Whereas protective factors included a greater degree of perceived maternal warmth, and higher maternal education level coupled with their own report of greater happiness in mid-childhood.

### History of traumatic events

Regarding traumatic events that were experienced in childhood ( Table 4), there were two that were linked to increased risk of externality (death of a friend and serious accident of a parent). In contrast, increased likelihood of internality was associated with the death of a relative, a parent or a sibling being in hospital and the mental illness of a parent. We suggest that these contrasting scenarios can be explained, in part, by increasing externality being associated with sudden unexpected events, which the women could have had no influence over, whereas observation of chronic illnesses and how others cope with them may provide models and experiences helpful in becoming more internal.

### Time and place

There was clear evidence that the more recently the women’s parents had been born the greater the risk of ELOC orientation. We have indicated in our earlier study that this finding fits with the trend of increasing ELOC rates over time, and we have shown that this does not appear to be explained by maternal age effects ( Golding *et al.*, 2017). The study was concerned with the women’s parents (born between 1890 and 1975), during which time the general environmental living conditions were improving and the education levels of the population were increasing. It is difficult to know how these features may have influenced an increase in external orientation unless one suggests that the struggle of families to survive and thrive was not so critical, especially after the Second World War in Britain; struggle in itself may have had a benefit in increasing internality.

One might suggest a similar explanation for the findings with migration and boarding school. The level of ELOC was markedly increased if a woman had been born in the same area in which she was living at the time her LOC was measured; most such women will not have moved from the area throughout their lives, and consequently will not have had the taxing task of changing from one area to another with all the likely changes in culture with which to adjust. Similarly girls who had been sent to boarding school, and thus away from their homes, would have had to develop strategies during childhood to increase their internality. It is also possible they were exposed to a greater variety of adult models of internality in their school and living environments.

### Relationship with previous research on antecedents of ELOC

The results of the present study identified a number of factors from mid- and late childhood associated with the presence of external locus of control in women expecting a child. Although we used an exposome approach with no predetermined theoretical predictions, many of the findings are consistent with what Carton & Nowicki (1994) concluded after their review of antecedent research. 

Children with generalized internal, as opposed to external, control expectancies report less stress earlier in their lives and have parents who report treating them more consistently, granting them greater autonomy to pursue their activities earlier, and providing them with a warm, supportive relationship. These associations have been found in data gathered from both males and females, ranging in age from 3 to 40 years. Although most of the findings have been obtained through self-report questionnaires, observational data, when obtained, often have provided important collaborative evidence. (p. 139).

However, they went on to criticize, among other things, previous research which was characterized by small, homogeneous samples. They urged researchers to gather data from larger prospective and longitudinal studies to evaluate whether the antecedents they had identified based on past research would also be found in more representative populations. The results of the present study regarding the importance of parents, especially parental “warmth” and the presence of parents in the life of their child, are consistent with past research findings and support Carton and Nowicki’s conclusions.

Finding that a lack of parental warmth and presence is associated with the development of external locus of control is consistent with the theorizing of Rotter (1966) and Lefcourt (1976) who believed that if children did not have a safe and secure social environment that supported “contingent” learning of the connection between their behavior and its consequences, they would fail to recognize their role in determining what happens to them. That description is consistent with our findings for women.

As well as substantiating the importance of parents’ ability to create a safe, secure learning environment, the present study also identified other antecedents that have not received much attention from past researchers. Two of the most significant are the (1) degree to which parents and children smoke and (2) the timing and type of traumatic experience. It could be that the smoking habits of those around them suggested a lack of understanding of the consequences of their actions in the future. Besides smoking behavior, traumatic experiences depending on their type and timing, were found to be associated at times with external and at other times with internal locus of control. Previous research and theory would suggest that the experience of trauma is associated with externality. However, in the present study we found externality to be linked only to trauma that could be described as “unexpected” (death of a peer, a friend) while internality was associated with trauma that was more characterized by “chronicity” (mental illness of a parent). These results suggest that how and when traumatic events occur and how they are perceived not only by the child, but also by those around the child, can facilitate the development of either internal or external control in children. Certainly, this process needs to be clarified by researchers so that we can more fully understand how to help children deal with trauma in a positive manner regarding the development of internal locus of control.

### Strengths and limitations

This study has major strengths in its size (considerably larger than any other studies of LOC in women), the detailed information it has on the women’s reports of their childhood and their parents, and the ability to link with earlier findings from this cohort ( Golding *et al.*, 2017).

The limitations should, however, be kept in mind. Firstly, we may not have taken into account a key feature of the women’s childhood that may have had a profound effect on her LOC orientation. One possible example would be the LOC orientations of each of her parents. Secondly, although ALSPAC is a population study, participation was voluntary, and an estimated 20% of the population did not take part; it is likely that these were weighted with women who had an ELOC. Thirdly, the information collected about the past was, by its very nature, obtained retrospectively.

The latter drawbacks will be addressed in later studies from this cohort of parents and their children, as we will be able to ascertain the ways in which the child’s LOC orientation is related to that of each parent. We will be able to chart the various social environments and traumatic events that have occurred, since we have identified them prospectively, and we will be able to assess the sizes of bias due to failure to follow-up.

## Conclusions

The Avon Longitudinal Study of Parents and Children (ALSPAC) has the benefit of having collected LOC information from parents before their child was born. The wealth of data on the women’s backgrounds and history, including their own childhoods, has provided information for investigation of historical factors that influence the development of an external orientation in these women. As a preliminary to documenting the way in which children develop their own LOCs, in this study we have used the facets of each pregnant woman’s childhood to assess which features appear predominant in the development of an ELOC. In particular, the data can be used to query received assumptions as to the origins of ELOC and to raise new hypotheses.

The results for these adult women stress the following for the development of internal versus external control: (i) the importance of childhood, particularly mid-childhood, between ages 6–11; (ii) the unexpected associations with cigarette smoking, both from prenatal exposure and from commencing regular smoking mid-childhood; (iii) the revelation that some stressors are more predictive of internality whereas others are of externality; and (iv) the risk of ELOC appears to rise dramatically for women whose parents were born during and after the Second World War.

Clearly, these results need to be confirmed in other populations. With the ALSPAC study, we will determine whether the same associations are apparent in men, and we will ascertain in more detail in the future which antecedent factors predict ELOC in the late teenage years.

## Data availability

The data referenced by this article are under copyright with the following copyright statement: Copyright: © 2017 Golding J et al.

In order to preserve confidentiality of the participants it is important that the ALSPAC access rules are taken into account. The ALSPAC study website contains details of all the data that are available through a fully searchable data dictionary: http://www.bris.ac.uk/alspac/researchers/data-access/data-dictionary/.

Data can be obtained by bona fide researchers after application to the ALSPAC Executive Committee ( http://www.bristol.ac.uk/alspac/researchers/access/).

## Supplementary material

**Supplementary File 1. Details of the antecedent variables considered in the study.**

**Supplementary Tables 1 – 10.**

Supplementary Table 1. Backwards step-wise logistic regression of the women’s locus of control score (>4 versus ≤4): early childhood (<6 years).

Supplementary Table 2. Backwards step-wise logistic regression of the women’s locus of control score (>4 versus ≤4): mid childhood

Supplementary Table 3. Backwards step-wise logistic regression of the women’s locus of control score (>4 versus ≤4): adolescence

Supplementary Table 4. Unadjusted associations between proportion of women with ELOC and childhood social care

Supplementary Table 5. Backwards step-wise logistic regression of the women’s locus of control score (>4 versus ≤4): social environment in childhood.

Supplementary Table 6. Unadjusted associations between proportion of women with ELOC and experience of life events in childhood

Supplementary Table 7. Backwards step-wise logistic regression of the women’s locus of control score (>4 versus ≤4): Life events in her childhood (excluding becoming pregnant, which may have been influenced by her own LOC)

Supplementary Table 8. Backwards stepwise logistic regression of the women’s ELOC score: her childhood and adolescence, life events and school & home environment

Supplementary Table 9. Backwards stepwise logistic regression of the women’s locus of control score (>4 versus ≤4): characteristics of her parents.

Supplementary Table 10. Backwards step-wise logistic regression of women’s locus of control score (>4 versus ≤4): their childhood and adolescence, life events, social care and characteristics of their parents.
