# Supplementary material for: The mid-childhood and adolescent antecedents of women’s external locus of control orientation
Source: Wellcome Open Res. 2017 Nov 6;2:53. Originally published 2017 Jul 21. [Version 2] doi: 10.12688/wellcomeopenres.12052.2 (PMC5710168; doi:10.12688/wellcomeopenres.12052.2)
Supplement: Supplementary file 1 [file wellcomeopenres-2-14254-s0000.tgz › 93743d41-6461-4346-bd8b-8579be11e18f.pdf]

Supplementary File 1. Details of the antecedent variables considered in the study.

Data relevant to the parents of the women.

The questionnaire asked about specific medical histories of the natural mother and of the mother figure (where relevant), including details of psychiatric and medical conditions. Similar data were collected for the father or father-figure. The 14 specific questions were: insulin dependent diabetes, non-insulin dependent diabetes, coronary heart disease, rheumatism, arthritis, multiple sclerosis, breast cancer (mothers) or prostate cancer (fathers), other type of cancer, hypertension, alcohol problem, schizophrenia, chronic bronchitis, stroke, and depression. A further question asked whether there were any other problems, and if so to describe them. Information on death of a parent and age at which the parent died was also requested, together with information on who cared for the study woman after the death.

Education

Information was obtained on all the qualifications of the women, their own mothers and their own fathers. From the information obtained, a 5-point education scale has been obtained for each, with the following categories: no. of qualifications obtained; no. of qualifications higher than CSE or GCSE (D, E, F or G); O-level or equivalent; A-level or equivalent; teaching or nursing qualification; University degree. This scale was similar to that derived for the Child Health & Education Study (Osborn *et al*. 1984).

Parental occupation

Data were collected for each parent on type of employment(s) comprising their usual job role, occupation, profession or trade, the industry in which they worked or the service(s) given. Occupations were classified using the SOC (Standard Occupational Classification) codes, which divides occupations into nine major groups based upon the qualifications and skills necessary to perform each job optimally. SOC is published by the Employment Department Group Office of Population Censuses and Surveys of Great Britain (OPCS) and the version used was published in 1990.

Ethnic origins

The ethnic origins of both the women and their parents were obtained using the format used in the 1991 United Kingdom Census. This categorizes the individual as White, Black/Caribbean, Black/African, Black/Other, Indian, Pakistani, Bangladeshi, Chinese, Other Specified.

Data relevant to experiences of childhood.

Home stability

The women were asked to rate the stability and predictability of each parent's behavior during their childhood. The question was: ‘was your parent’s behavior stable and predictable to you as a child’? With possibly answers: always, mostly, rarely, never. The data were obtained for mother, father, mother figure, father figure, and a ‘home stability’ variable derived from the answers. These questions were created by Karen Thorpe, specifically for ALSPAC.

Relationship with the mother

The relationship of the women with their own mother (or mother figure) was defined using a 22-item set of questions modified from the original Parent-Bonding Instrument (PBI). Respondents reported the quality of the relationship with their mother in childhood on two scales: the care and over protectiveness subscales. (Parker *et al*. 1979). Previous research supports the validity and reliability of this scale, particularly the validity of retrospective reports (Parker, 1981). The original Parental Bonding Instrument (Parker *et al*. 1979) had been adapted by Gamsa (1987) to reword the statements that had produced double negatives in the original. Over the course of piloting, it became obvious that our parents were unhappy with the original options for responses (very like, moderately like, moderately unlike, very unlike) and they have been changed to: 'never', 'sometimes', and 'usually'. In addition, three questions were omitted since they were almost identical to other questions in the scale and caused considerable annoyance to participants. The introduction to the 22 statements read as follows: "We would like to know how you and your mother got on when you were a child. This will probably have varied over your childhood and in different situations but we would like a general impression. Please tick the box to indicate how you mostly remember your mother in the first 16 years". Two scores were derived from these 22 questions: a 'maternal care' score and an 'overprotective' score. Internal consistencies in this sample were .73 and .70 for care and over-protectiveness, respectively (O'Connor *et al*. 1999).

Childhood happiness

Also developed by Karen Thorpe, the questions to the women were worded: "Looking back would you call your childhood happy?" For each of the ages 0-5, 6-11, 12-15 she was given options 'yes very happy', 'yes moderately happy', 'not really happy', 'no quite unhappy', 'no very unhappy'. After these questions, there was space for any comments the mothers may have wanted to add. From the answers to the three questions, a variable concerning childhood happiness was derived to distinguish those who were very happy throughout.

Other data relevant to the study women's childhoods

This section identified mothers who had been adopted, in care, or who had experienced a number of other events during their childhood. All respondents reported the living arrangements for three intervals: 0-5 years, 6-11 years, and 12-16 years. It asked the age at which parents divorced or separated, and child care of the mother after this, periods when lived with other relatives, friends or foster-parents, including in boarding school, in a hostel, in custody, etc. It elicited the composition of the household at different ages during childhood. For each interval, respondents reported whether they were living with a biological or step mother and father, or were in other arrangements (e.g. living with grandparents). Thus, from these reports it was possible to assess, to a limited degree, whether individuals lived in a stepfamily following parental separation, and the degree of disruption that followed the separation. As noted by O'Connor *et al* (1999), it was not possible to differentiate those who experienced two or more transitions within the same approximate 5-year window.

Childhood life events

This scale comprised a set of questions of 31 specific items; it was administered to the pregnant women in the third trimester of pregnancy. Childhood was specified as being <17 years. The items were devised by the ALSPAC Study Team based on the earlier work of Coddington (1972) and included four on deaths to parent(s), relative(s), sibling(s) and

friend(s); three on serious illness to the participant as well as to a parent and sibling; three on experiencing a serious accident (to parent, participant, sibling); three on hospitalization (to parent, participant and sibling); three concerning abuse to the participant (physical, sexual and emotional); seven relating to parents (separated, divorced, had serious arguments, remarried, imprisoned, mentally ill, family became poorer); and seven to the participants themselves (discovered that they were adopted, failed an important exam, moved to a new district, in trouble with the police, expelled or suspended from school, became physically deformed, became pregnant).

References

Coddington, R.D. The significance of life events as etiologic factors in the diseases of children. I. A survey of professional workers. *J Psychosomat Res.* 1972; **16** (1): 7-18. Doi. 10.1016/0022-3999(72)90018-9

Gamsa, A. A note on a modification of the Parent Bonding Instrument. *Br J Med Psychol.* 1987; **60**:291-294. Doi. 10.1111/j.2044-8341.1987.tb02745.x

O`Connor, T.G., Thorpe, K., Dunn, J., Golding, J. Parental divorce and adjustment in adulthood: findings from a community sample. The ALSPAC Study Team. Avon Longitudinal Study of Pregnancy and Childhood. *J Child Psychol Psychiatr.* 1999; **40** (5): 777-89. Doi. 10.1111/1469-7610.00493

Office of Population Censuses and Surveys. *Standard Occupational Classification 1990.* London; Office of Population Censuses and Surveys. 1990.

Osborn A.F., Butler N.R., Morris A.C. *The Social Life of Britain's Five Year Olds. A Report of the Child Health & Education Study*. London: Routledge & Kegan Paul. 1984.

Parker, G. Parental reports of depressives: An investigation of several explanations. *J Affect Disord.* 1981; **3**: 131-140. Doi. 10.1016/0165-0327(81)90038-0.

Parker, G., Tupling, H., & Brown, L. B. A Parental Bonding Instrument. *Br J Med Psychol.* 1979; **52**:1-10. DOI: 11111/ji.2044-8341.1979.tb02487.x
