# Supplementary material for: The mid-childhood and adolescent antecedents of women’s external locus of control orientation
Source: Wellcome Open Res. 2017 Nov 6;2:53. Originally published 2017 Jul 21. [Version 2] doi: 10.12688/wellcomeopenres.12052.2 (PMC5710168; doi:10.12688/wellcomeopenres.12052.2)
Supplement: Supplementary file 2 [file wellcomeopenres-2-14254-s0001.tgz › 90793b75-cf0d-4cb2-b263-749b5f78481b.pdf]

Supplementary Table 1. Backwards step-wise logistic regression of the women's locus of control score (>4 versus ≤4): early childhood (<6 years)

	Univariable			Intra domain		
**Features of her early childhood**	**N**	**P**	**OR [95% CI]**	**N**	**P**	**OR [95% CI]**
Has birthmark	12638	<0.0001	1.23 [1.13, 1.33]	8614	0.002	1.18 [1.06, 1.30]
Year of birth	12564	<0.0001	1.79 [1.70, 1.89]	8614	<0.0001	1.65 [1.54, 1.77]
Born in Avon	11534	<0.0001	2.06 [1.91, 2.22]	8614	<0.0001	1.80 [1.64, 1.97]
Was breast fed	10007	<0.0001	0.73 [0.67, 0.79]	8614	<0.001	0.85 [0.78, 0.93]
Number of older siblings	11937	<0.0001	1.33 [1.22, 1.44]	8614	<0.0001	1.27 [1.13, 1.41]
Mother present in household	12638	<0.0001	0.44 [0.38, 0.52]	8614	0.072	0.74 [0.54, 1.03]
Father present in household	12638	<0.0001	0.43 [0.38, 0.50]	8614	<0.0001	0.53 [0.44, 0.64]
Stepfather present in household	12638	0.006	1.60 [1.14, 2.23]	8614	0.156	0.72 [0.45, 1.14]
Step-siblings present in household	12638	0.004	1.69 [1.18, 2.43]	8614	0.564	1.17 [0.68, 2.02]
Mother died	12637	<0.0001	3.08 [1.75, 5.40]	8614	0.218	1.84 [0.70, 4.85]
Parents divorced/separated	12424	<0.0001	2.09 [1.77, 2.46]	8506	0.670	1.05 [0.83, 1.34]
Unhappiness in early childhood	12637	<0.0001	3.08 [1.75, 5.40]	8614	0.218	0.54 [0.21, 1.43]

GOF = 5.79

Supplementary Table 2. Backwards step-wise logistic regression of the women's locus of control score (>4 versus ≤4): mid childhood

	Univariable			Intra domain		
**Features of her mid-childhood**	**N**	**P**	**OR [95% CI]**	**N**	**P**	**OR [95% CI]**
Mother present in household	12638	<0.0001****	0.45 [0.39, 0.52]	12090	<0.0001****	0.59 [0.50, 0.70]
Father present in household	12638	<0.0001****	0.52 [0.47, 0.58]	12090	<0.0001****	0.71 [0.63, 0.81]
Stepfather present in household	12638	<0.0001****	1.49 [1.24, 1.79]	12090	0.416	0.91 [0.73, 1.14]
Step-siblings present in household	12638	<0.0001****	1.82 [1.41, 2.36]	12090	0.108	1.25 [0.95, 1.65]
Mother's partner present in household	12638	<0.0001****	1.94 [1.40, 2.70]	12090	0.175	1.27 [0.90, 1.80]
Parents divorced/separated	12424	<0.0001****	1.54 [1.33, 1.78]	11913	0.144	1.13 [0.96, 1.32]
Unhappiness in mid childhood	12529	<0.0001****	1.35 [1.28, 1.41]	12090	<0.0001****	0.79 [0.75, 0.83]
Smoked regularly by age 11	12184	<0.0001****	3.59 [2.53, 5.12]	12090	<0.0001****	3.16 [2.19, 4.58]
Menarche occurred	12635	0.006**	1.14 [1.04, 1.24]	12090	0.038*	1.11 [1.01, 1.22]

GOF = 1.99

Supplementary Table 3. Backwards step-wise logistic regression of the women's locus of control score (>4 versus ≤4): adolescence

	Univariable			Intra domain		
**Features of her adolescence**	**N**	**P**	**OR [95% CI]**	**N**	**P**	**OR [95% CI]**
Mother absent from household	12633	<0.0001****	1.93 [1.68, 2.22]	12574	<0.0001****	1.62 [1.40, 1.88]
Father absent from household	12633	<0.0001****	1.66 [1.51, 1.81]	12574	<0.0001****	1.44 [1.31, 1.59]
Stepfather present in household	12633	<0.0001****	1.38 [1.19, 1.60]	12574	0.635	0.96 [0.80, 1.14]
Step-brother present in household	12633	<0.001***	1.57 [1.22, 2.01]	12574	0.598	1.07 [0.83, 1.39]
Step-sister present in household	12633	<0.001***	1.71 [1.30, 2.25]	12574	0.222	1.19 [0.90, 1.57]
Mother's partner present in household	12633	<0.0001****	1.68 [1.30, 2.18]	12574	0.146	1.22 [0.93, 1.60]
Parents divorced/separated	12419	<0.001***	0.74 [0.62, 0.88]	12380	0.962	1.00 [0.84, 1.21]
Happiness in adolescence	12574	<0.0001****	0.85 [0.82, 0.88]	12574	<0.0001****	0.89 [0.86, 0.92]

GOF = 1.26

Supplementary Table 4. Unadjusted associations between proportion of women with ELOC and childhood social care

Social environment in childhood	%(n) ELOC	OR [95% CI]	P
Attended a special school	N=12638		<0.0001
Yes	69.2% (126)	2.76 [2.01, 3.79]	
No	44.9% (5592)	1.00 Ref	
Saw a child psychiatrist	N=12638		<0.0001
Yes	59.3% (278)	1.80 [1.49, 2.17]	
No	44.7% (5440)	1.00 Ref	
Had speech therapy	N = 12638		0.001
Yes	54.4% (161)	1.46 [1.16, 1.84]	
No	45.9% (2275)	1.00 Ref	
Was in care	N = 12012		<0.0001
Yes	69.0% (185)	2.76 [2.13, 3.59]	
No	44.6% (5242)	1.00 Ref	
Lived with grandparents	N=12480		<0.0001
Yes	60.8% (401)	1.94 [1.65, 2.27]	
No	44.4% (5250)	1.00 Ref	
Lived with other relatives	N=12480		<0.001
Yes	55.4% (275)	1.53 [1.28, 1.83]	
No	44.9% (5376)	1.00 Ref	
Lived with friends	N=12480		<0.0001
Yes	61.2% (289)	1.96 [1.62, 2.36]	
No	44.7% (5362)	1.00 Ref	
Lived with foster parents	N=12479		<0.0001
Yes	67.3% (109)	2.51 [1.81, 3.50]	
No	45.0% (5541)	1.00 Ref	
Went to boarding school	N=12383		<0.0001
Yes	25.9% (121)	0.41 [0.33, 0.51]	
No	45.9% (5465)	1.00 Ref	
Stayed in children's home	N=12374		<0.0001
Yes	66.8% (133)	2.48 [1.84, 3.34]	
No	44.8% (5454)	1.00 Ref	
Left home before age 18	N=12489		<0.0001
Yes	56.8% (1415)	1.78 [1.63, 1.95]	
No	42.4% (4239)	1.00 Ref	
Stability of mother in household	N=12217		<0.0001
Always	43.3% (3002)	1.00 Ref	
Mostly	45.5% (2099)	1.09 [1.01, 1.18]	
Rarely / never	60.0% (396)	1.97 [1.67, 2.32]	

Stability of father in household	N=11650		<0.0001
Always	42.5% (2715)	1.00 Ref	
Mostly	45.1% (1912)	1.11 [1.03, 1.20]	
Rarely /never	53.8% (555)	1.57 [1.38, 1.79]	
Overall stability of home	N=12420		<0.0001
Very stable	43.2% (2436)	1.00 Ref	
Fairly stable	44.5% (2324)	1.05 [0.98, 1.14]	
Unstable	53.1% (569)	1.49 [1.31, 1.70]	
Very unstable	57.2% (275)	1.76 [1.46, 2.12]	
Maternal care score	N=12637		<0.0001
<19	52.1% (1762)	1.77 [1.65, 1.89]	
19-21	48.4% (1185)	1.52 [1.37, 1.69]	
22-23	43.2% (1488)	1.23 [1.12, 1.36]	
24	38.1% (1282)	1.00 Ref	

Supplementary Table 5. Backwards step-wise logistic regression of the women's locus of control score (>4 versus ≤4): social environment in childhood.

	Univariable			Intra domain		
**Variable**	**N**	**P**	**OR [95% CI]**	**N**	**P**	**OR [95% CI]**
Attended special school	12638	<0.0001****	2.76 [2.01, 3.79]	10851	<0.0001****	2.54 [1.75, 3.69]
Saw physiotherapist	12638	0.004**	0.80 [0.69, 0.93]	10851	<0.001***	0.73 [0.62, 0.86]
Attended child psychiatrist	12638	<0.0001****	1.80 [1.49, 2.17]	10851	0.004**	1.39 [1.11, 1.73]
Had speech therapy	12638	0.001**	1.46 [1.16, 1.84]	10851	0.110	1.24 [0.95, 1.63]
In care	12012	<0.0001****	2.76 [2.13, 3.59]	10851	<0.001***	2.11 [1.43, 3.11]
Lived with grandparents	12480	<0.0001****	1.94 [1.65, 2.27]	10851	<0.0001****	1.52 [1.24, 1.86]
Lived with other relatives	12480	<0.0001****	1.53 [1.28, 1.83]	10851	0.513	1.08 [0.86, 1.35]
Lived with friends	12480	<0.0001****	1.96 [1.62, 2.36]	10851	0.010*	1.34 [1.07, 1.68]
Lived with foster parents	12479	<0.0001****	2.51 [1.81, 3.50]	10851	0.076	1.76 [0.94, 3.27]
Went to boarding school	12383	<0.0001****	0.41 [0.33, 0.51]	10851	<0.0001****	0.31 [0.24, 0.39]
Stayed in children's home	12374	<0.0001****	2.48 [1.84, 3.34]	10823	0.583	0.83 [0.42, 1.63]
Left home before age 18	12489	<0.0001****	1.78 [1.63, 1.95]	10851	<0.0001****	1.49 [1.34, 1.66]
Unstable/unpredictable mother	12217	<0.0001****	1.90 [1.62, 2.23]	10786	0.296	1.11 [0.91, 1.36]
Unstable/unpredictable father	11650	<0.0001****	1.51 [1.33, 1.71]	10851	0.024*	1.18 [1.02, 1.36]
Stayed in other place	12548	0.004**	0.80 [0.68, 0.93]	10851	<0.0001****	0.65 [0.55, 0.78]
Maternal care score	12637	<0.0001****	0.72 [0.68, 0.75]	10851	<0.0001****	0.77 [0.73, 0.82]
Home unstable	12420	<0.0001****	1.36 [1.26, 1.47]	10851	0.905	0.99 [0.85, 1.15]

GOF = 2.84

Supplementary Table 6. Unadjusted associations between proportion of women with ELOC and experience of life events in childhood

Experiences in childhood	%(n) ELOC	OR [95% CI]	P
A parent died	N=11843		0.283
Yes	46.5% (328)	1.10 [0.94, 1.28]	
No	44.2% (4923)	1.00 Ref	
A sibling died	N=11843		0.900
Yes	44.0% (124)	0.98 [0.78, 1.25]	
No	44.3% (5127)	1.00 Ref	
A relative died	N = 11843		<0.0001
Yes	41.2% (2588)	0.76 [0.71, 0.82]	
No	47.9% (2663)	1.00 Ref	
A friend died	N = 11843		0.002
Yes	47.9% (786)	1.18 [1.06, 1.31]	
No	43.8% (4465)	1.00 Ref	
Parent seriously ill	N=11843		0.017
Yes	42.0% (885)	0.89 [0.81, 0.98]	
No	44.8% (4366)	1.00 Ref	
Parent in hospital	N=11843		<0.0001
Yes	40.9% (1881)	0.79 [0.74, 0.85]	
No	46.6% (3370)	1.00 Ref	
Was seriously ill	N=11843		0.073
Yes	40.7% (229)	0.85 [0.72, 1.01]	
No	44.5% (5022)	1.00 Ref	
Was admitted to hospital	N=11843		<0.001
Yes	42.0% (1655)	0.87 [0.80, 0.94]	
No	45.5% (3596)	1.00 Ref	
A sibling was seriously ill	N=11843		0.006
Yes	39.9% (359)	0.82 [0.72, 0.94]	
No	44.7% (4892)	1.00 Ref	
A sibling was in hospital	N=11843		<0.0001
Yes	39.8% (999)	0.79 [0.72, 0.86]	
No	45.6% (4252)	1.00 Ref	
Parent had a serious accident	N=11843		0.002
Yes	51.1% (254)	1.33 [1.11, 1.59]	
No	44.0% (4997)	1.00 Ref	
Had a serious accident	N=11843		0.952
Yes	44.5% (241)	1.01 [0.85, 1.20]	
No	44.3% (5010)	1.00 Ref	

Became pregnant	N=11843		<0.0001
Yes	64.5% (557)	2.44 [2.11,2.82]	
No	42.8% (4694)	1.00 Ref	
A parent was imprisoned	N = 11843		0.255
Yes	50.6% (41)	1.29 [0.83, 2.00]	
No	44.3% (5210)	1.00 Ref	
Was physically abused by parent	N = 11843		0.001
Yes	52.4% (208)	1.40 [1.14, 1.71]	
No	44.1% (5043)	1.00 Ref	
Parents separated	N=11843		<0.0001
Yes	53.2% (1056)	1.53 [1.39, 1.69]	
No	42.6% (4195)	1.00 Ref	
Parents divorced	N=11843		<0.0001
Yes	55.8% (999)	1.73 [1.56, 1.91]	
No	42.3% (4252)	1.00 Ref	
A parent remarried	N=11843		<0.0001
Yes	53.6% (725)	1.52 [1.36, 1.71]	
No	43.1% (4526)	1.00 Ref	
Was emotionally abused by parent	N = 11843		0.035
Yes	47.7% (432)	1.16 [1.01, 1.33]	
No	44.1% (4819)	1.00 Ref	
Parents had serious arguments	N = 11843		0.004
Yes	46.5% (1469)	1.13 [1.04, 1.22]	
No	43.5% (3782)	1.00 Ref	
Was sexually abused	N=11843		<0.0001
Yes	53.2% (318)	1.45 [1.23, 1.71]	
No	43.9% (4933)	1.00 Ref	
A parent was mentally ill	N = 11843		<0.001
Yes	36.4% (186)	0.71 [0.59, 0.85]	
No	44.7% (5065)	1.00 Ref	
Discovered was adopted	N=11843		0.069
Yes	50.2% (117)	1.27 [0.98, 1.65]	
No	44.2% (4959)	1.00 Ref	
Moved to a new district	N=11843		<0.0001
Yes	39.6% (1274)	0.77 [0.70, 0.83]	
No	46.1% (3977)	1.00 Ref	
In trouble with police	N=11843		<0.0001
Yes	64.2% (292)	2.32 [1.91, 2.82]	
No	43.5% (4959)	1.00 Ref	

Was suspended from school	N = 11843		<0.0001
Yes	65.5% (264)	2.46 [1.99, 3.03]	
No	43.6% (4987)	1.00 Ref	
Family finances deteriorated	N = 11843		0.426
Yes	45.3% (670)	1.05 [0.94, 1.17]	
No	44.2% (4581)	1.00 Ref	

Supplementary Table 7. Backwards step-wise logistic regression of the women's locus of control score (>4 versus ≤4): Life events in her childhood (excluding becoming pregnant, which may have been influenced by her own LOC).

	Univariable			Intra domain		
**Experience in childhood**	**N**	**P**	**OR [95% CI]**	**N**	**P**	**OR [95% CI]**
Relative died	11843	<0.0001****	0.76 [0.71, 0.82]	11843	<0.0001****	0.80 [0.74, 0.86]
Friend died	11843	0.002**	1.18 [1.06, 1.31]	11843	<0.0001****	1.28 [1.15, 1.42]
Parent seriously ill	11843	0.017*	0.89 [0.81, 0.98]	11843	0.738	1.02 [0.91, 1.14]
Parent in hospital	11843	<0.0001****	0.79 [0.74, 0.85]	11843	<0.0001****	0.84 [0.78, 0.91]
Admitted to hospital	11843	<0.001***	0.87 [0.80, 0.94]	11843	0.058	0.92 [0.85, 1.00]
Sibling seriously ill	11843	0.006**	0.82 [0.72, 0.94]	11843	0.408	0.94 [0.80, 1.10]
Sibling in hospital	11843	<0.0001****	0.79 [0.72, 0.86]	11843	<0.0001****	0.83 [0.75, 0.91]
Parent had serious accident	11843	0.002**	1.33 [1.11, 1.59]	11843	<0.0001****	1.51 [1.26, 1.82]
Physically abused by parent	11843	0.001**	1.40 [1.14, 1.71]	11843	0.032*	1.27 [1.02, 1.57]
Parents separated	11843	<0.0001****	1.53 [1.39, 1.69]	11843	0.884	1.01 [0.84, 1.22]
Parents divorced	11843	<0.0001****	1.73 [1.56, 1.91]	11843	<0.0001****	1.72 [1.55, 1.91]
Parents remarried	11843	<0.0001****	1.52 [1.36, 1.71]	11843	0.911	1.01 [0.86, 1.19]
Emotionally abused by parent	11843	0.035*	1.16 [1.01, 1.33]	11843	0.981	1.00 [0.85, 1.18]
Parents had serious arguments	11843	0.004**	1.13 [1.04, 1.22]	11843	0.294	1.05 [0.96, 1.15]
Sexually abused	11843	<0.0001****	1.45 [1.23, 1.71]	11843	<0.001***	1.38 [1.16, 1.64]
Parent mentally ill	11843	<0.001***	0.71 [0.59, 0.85]	11843	<0.0001****	0.65 [0.54, 0.79]
Moved to new district	11843	<0.0001****	0.77 [0.70, 0.83]	11843	<0.0001****	0.75 [0.68, 0.81]
Acquired step-sibling	11843	<0.0001****	1.46 [1.28, 1.68]	11843	0.779	1.02 [0.87, 1.21]

GOF = 1.94

Supplementary Table 8. Backwards stepwise logistic regression of the woman's ELOC score: her childhood and adolescence, life events and school & home environment

	Univariable			Intra domain		
**Variable**	**N**	**P**	**OR [95% CI]**	**N**	**P**	**OR [95% CI]**
In early childhood						
Has birthmark	12633	<0.0001****	1.23 [1.13, 1.33]	8673	0.001**	1.19 [1.07, 1.32]
Year of birth	12559	<0.0001****	1.79 [1.70, 1.89]	8673	<0.0001****	1.53 [1.43, 1.65]
Born in Avon	11531	<0.0001****	2.06 [1.91, 2.22]	8673	<0.0001****	1.81 [1.65, 1.99]
Breast fed	10003	<0.0001****	0.73 [0.67, 0.79]	8673	0.003**	0.87 [0.79, 0.96]
Number of older siblings	11932	<0.0001****	1.33 [1.22, 1.45]	8673	<0.0001****	1.26 [1.12, 1.41]
Father absent from household	12633	<0.0001****	2.30 [2.01, 2.62]	8673	<0.0001****	1.61 [1.32, 1.97]
In mid-childhood						
Mother absent from household	12633	<0.0001****	2.22 [1.93, 2.55]	8673	0.271	1.18 [0.88, 1.60]
Father absent from household	12633	<0.0001****	1.90 [1.72, 2.11]	8673	0.675	0.96 [0.80, 1.16]
Happiness in mid childhood	12524	<0.0001****	0.74 [0.71, 0.78]	8673	0.001**	0.88 [0.81, 0.95]
Smoked regularly	12179	<0.0001****	3.60 [2.53, 5.12]	8673	0.027*	1.72 [1.06, 2.78]
Menarche before age 12	12630	0.006**	1.14 [1.04, 1.25]	8671	0.667	1.03 [0.91, 1.15]
In adolescence						
Mother absent from household	12633	<0.0001****	1.93 [1.68, 2.22]	8673	0.046*	1.26 [1.00, 1.58]
Father absent from household	12633	<0.0001****	1.66 [1.51, 1.81]	8673	0.908	1.01 [0.88, 1.16]
Happiness in adolescence	12574	<0.0001****	0.85 [0.82, 0.88]	8673	0.422	0.97 [0.91, 1.04]
Traumatic life events						
Relative died	11838	<0.0001****	0.76 [0.71, 0.82]	8673	<0.001***	0.85 [0.77, 0.93]
Friend died	11838	0.002**	1.18 [1.06, 1.31]	8673	0.004**	1.21 [1.07, 1.39]
Parent in hospital	11838	<0.0001****	0.79 [0.74, 0.86]	8673	0.038*	0.90 [0.82, 0.99]
Sibling in hospital	11838	<0.0001****	0.79 [0.72, 0.86]	8673	0.003**	0.84 [0.75, 0.94]
Parent had serious accident	11838	0.002**	1.33 [1.11, 1.59]	8673	0.001**	1.45 [1.16, 1.82]
Physically abused by parent	11838	0.001**	1.40 [1.14, 1.71]	8673	0.034*	0.73 [0.55, 0.98]
Parents divorced	11838	<0.0001****	1.73 [1.56, 1.91]	8673	0.491	1.05 [0.91, 1.21]
Sexually abused	11838	<0.0001****	1.45 [1.23, 1.71]	8673	0.694	1.05 [0.84, 1.30]
Parent mentally ill	11838	<0.001***	0.71 [0.59, 0.85]	8673	<0.001***	0.65 [0.51, 0.82]
Moved to new district	11838	<0.0001****	0.77 [0.71, 0.83]	8673	0.079	0.91 [0.81, 1.01]
Social care						

Attended special school	12633	<0.0001****	2.76 [2.01, 3.79]	8673	0.064	1.52 [0.98, 2.38]
Saw physiotherapist	12633	0.004**	0.80 [0.69, 0.93]	8673	0.207	0.89 [0.73, 1.07]
Attended child psychiatrist	12633	<0.0001****	1.80 [1.49, 2.17]	8673	0.017*	1.36 [1.06, 1.76]
In care	12007	<0.0001****	2.80 [2.15, 3.64]	8333	0.295	1.27 [0.81, 1.99]

Supplementary Table 9. Backwards stepwise logistic regression of the woman's locus of control score (>4 versus ≤4): characteristics of her parents.

	Univariable			Intra domain		
**Characteristics of her parents**	**N**	**P**	**OR [95% CI]**	**N**	**P**	**OR [95% CI]**
Mother's year of birth	10642	<0.0001****	1.43 [1.37, 1.48]	5975	<0.0001****	1.27 [1.14, 1.42]
Mother's education ≥ O-Level	11949	<0.0001****	0.47 [0.43, 0.51]	5975	<0.0001****	0.69 [0.60, 0.79]
Mother smoked when pregnant	12633	<0.0001****	1.63 [1.52, 1.76]	5975	<0.0001****	1.32 [1.17, 1.48]
Mother aged <25 at birth	10651	<0.0001****	1.49 [1.38, 1.61]	5975	0.013*	0.81 [0.69, 0.96]
Father's year of birth	9876	<0.0001****	1.38 [1.32, 1.43]	5975	<0.0001****	1.24 [1.12, 1.38]
Father's education ≥ O-Level	8422	<0.0001****	0.49 [0.45, 0.54]	5975	0.038*	0.86 [0.75, 0.99]
Father smoked	11621	<0.0001****	1.50 [1.37, 1.64]	5975	<0.001***	1.25 [1.09, 1.42]
Father aged <25 at birth	9885	<0.0001****	1.58 [1.44, 1.74]	5975	0.041*	0.82 [0.68, 0.99]
Father's social group	9632	<0.0001****	1.20 [1.17, 1.22]	5975	<0.0001****	1.14 [1.10, 1.18]

GOF = 5.74

Supplementary Table 10. Backwards step-wise logistic regression of women's locus of control score (>4 versus ≤4): their childhood and adolescence, life events, social care and characteristics of their parents.

	Univariable			Intra domain		
	**N**	**P**	**OR [95% CI]**	**N**	**P**	**OR [95% CI]**
In early childhood						
Had birthmark	12633	<0.0001****	1.23 [1.13, 1.33]	7285	0.158	1.09 [0.97, 1.22]
Year of birth	12559	<0.0001****	1.79 [1.70, 1.89]	7285	<0.0001****	1.38 [1.25, 1.52]
Born in Avon	11531	<0.0001****	2.06 [1.91, 2.22]	7285	<0.0001****	1.61 [1.44, 1.79]
Breast fed	10003	<0.0001****	0.73 [0.67, 0.79]	6356	0.272	0.94 [0.84, 1.05]
Number of older siblings	11932	<0.0001****	1.33 [1.22, 1.45]	7285	0.011*	1.19 [1.04, 1.35]
Father absent from household	12633	<0.0001****	2.30 [2.01, 2.62]	7285	0.019*	1.35 [1.05, 1.74]
Mother absent from household	12633	<0.0001****	2.22 [1.93, 2.55]	7285	0.503	1.11 [0.81, 1.52]
In mid childhood						
Father absent from household	12633	<0.0001****	1.90 [1.72, 2.11]	7285	0.704	1.05 [0.82, 1.33]
Happiness in mid childhood	12524	<0.0001****	0.74 [0.71, 0.78]	7285	0.018*	0.90 [0.83, 0.98]
Smoked regularly by 11	12179	<0.0001****	3.60 [2.53, 5.12]	7285	0.045*	1.78 [1.01, 3.12]
Menarche	12630	0.006**	1.14 [1.04, 1.25]	7284	0.900	1.01 [0.89, 1.15]
In adolescence						
Mother absent from household	12633	<0.0001****	1.93 [1.68, 2.22]	7285	0.901	1.02 [0.78, 1.32]
Father absent from household	12633	<0.0001****	1.66 [1.51, 1.81]	7285	0.528	1.06 [0.89, 1.26]
Happiness	12574	<0.0001****	0.85 [0.82, 0.88]	7285	0.894	1.00 [0.93, 1.08]
Traumatic life events						
Relative died	11838	<0.0001****	0.76 [0.71, 0.82]	7285	0.003**	0.86 [0.77, 0.95]
Friend died	11838	0.002**	1.18 [1.06, 1.31]	7285	0.008**	1.22 [1.05, 1.41]
Parent in hospital	11838	<0.0001****	0.79 [0.74, 0.86]	7285	0.087	0.91 [0.82, 1.01]
Sibling in hospital	11838	<0.0001****	0.79 [0.72, 0.86]	7285	0.005**	0.84 [0.74, 0.95]
Parent had serious accident	11838	0.002**	1.33 [1.11, 1.59]	7285	<0.001***	1.60 [1.25, 2.06]
Physically abused by parent	11838	0.001**	1.40 [1.14, 1.71]	7285	0.209	0.81 [0.58, 1.13]
Parents divorced	11838	<0.0001****	1.73 [1.56, 1.91]	7285	0.464	1.06 [0.90, 1.26]
Sexually abused	11838	<0.0001****	1.45 [1.23, 1.71]	7285	0.870	0.98 [0.76, 1.26]
Parent mentally ill	11838	<0.001***	0.71 [0.59, 0.85]	7285	0.001**	0.64 [0.48, 0.84]
Moved to new district	11838	<0.0001****	0.77 [0.71, 0.83]	7285	0.579	0.97 [0.86, 1.09]
Social care						

Attended special school	12633	<0.0001****	2.76 [2.01, 3.79]	7285	0.208	1.36 [0.84, 2.18]
Saw physiotherapist	12633	0.004**	0.80 [0.69, 0.93]	7285	0.669	0.95 [0.77, 1.18]
Attended child psychiatrist	12633	<0.0001****	1.80 [1.49, 2.17]	7285	0.138	1.25 [0.93, 1.68]
In care	12007	<0.0001****	2.80 [2.15, 3.64]	7285	0.011*	2.19 [1.19, 4.03]
Lived with grandparents	12475	<0.0001****	1.94 [1.65, 2.28]	7285	0.005**	1.45 [1.12, 1.89]
Lived with friends	12475	<0.0001****	1.96 [1.62, 2.36]	7285	0.439	1.13 [0.83, 1.55]
Went to boarding school	12378	<0.0001****	0.41 [0.33, 0.51]	7285	<0.001***	0.57 [0.42, 0.78]
Left home before age 18	12484	<0.0001****	1.79 [1.63, 1.95]	7285	<0.0001****	1.32 [1.15, 1.52]
Unstable/unpredictable father	11648	<0.0001****	1.51 [1.33, 1.71]	7094	0.896	1.01 [0.83, 1.25]
Stayed in other place	12543	0.004**	0.80 [0.68, 0.93]	7285	0.309	0.89 [0.72, 1.11]
Maternal care score	12632	<0.0001****	0.72 [0.68, 0.75]	7285	<0.0001****	0.79 [0.72, 0.86]
Features of parents						
Mother’s year of birth	10642	<0.0001****	1.43 [1.37, 1.48]	7285	<0.001***	1.14 [1.07, 1.21]
Mother’s education ≥ O-Level	11949	<0.0001****	0.47 [0.43, 0.51]	7285	<0.0001****	0.66 [0.59, 0.75]
Mother smoked when pregnant	12633	<0.0001****	1.63 [1.52, 1.76]	7285	0.005**	1.17 [1.05, 1.30]
Mother aged <25 at birth	10651	<0.0001****	1.49 [1.38, 1.61]	7285	0.597	0.96 [0.83, 1.11]
Father’s year of birth	9876	<0.0001****	1.38 [1.32, 1.43]	6976	0.814	0.99 [0.90, 1.08]
Father’s education ≥ O-Level	8422	<0.0001****	0.49 [0.45, 0.54]	5349	0.328	0.93 [0.80, 1.08]
Father ever smoked	11621	<0.0001****	1.50 [1.37, 1.64]	7285	<0.001***	1.23 [1.09, 1.39]
Father aged <25 at birth	9885	<0.0001****	1.58 [1.44, 1.74]	6976	0.758	0.98 [0.84, 1.13]
Father’s social group	9632	<0.0001****	1.20 [1.17, 1.22]	7285	<0.0001****	1.08 [1.05, 1.11]

GOF = 8.37
