# Supplementary material for: The mid-childhood and adolescent antecedents of women’s external locus of control orientation
Source: Wellcome Open Res. 2017 Nov 6;2:53. Originally published 2017 Jul 21. [Version 2] doi: 10.12688/wellcomeopenres.12052.2 (PMC5710168; doi:10.12688/wellcomeopenres.12052.2)
Supplement: Peer review history

# Peer review: Referee response for version 2

**DOI:** 10.21956/wellcomeopenres.14254.r27778

**Published:** 2017-11-27

**Authors:** Aleksandra Kostic

**Affiliations:** 1 Faculty of Philosophy, Study Group for Psychology, University of Niš, Niš, Serbia

This is an important study with many practical implications. I am satisfied with all of authors’ explanations. I understand their view and I can accept it. My compliments!

I have read this submission. I believe that I have an appropriate level of expertise to confirm that it is of an acceptable scientific standard.

# Peer review: Referee response for version 1

**DOI:** 10.21956/wellcomeopenres.13038.r25820

**Published:** 2017-10-18

**Authors:** Klaus A. Schneewind

**Affiliations:** 1 Department of Psychology, Ludwig Maximilian University of Munich, Munich, Germany

This is an important study highlighting the special influence of a theoretically and empirically psychological variable impacting on the health and well-being of human beings. Apart from the especially large sample the study addresses an important single variable relating to an appropriate development of human beings’ self-determination which has been shown in a broad range of national and international studies.

 Apart from an albeit large female sample the study would have profited from a cluster-analytic approach determining different constellations of predictive variables impacting on the subjects’ well-being. Moreover, a longitudinal approach to assessing the influence of external locus of control in combination with internal locus of control would allow to draw more specific influence on the subject’s well-being and potential to cope with challenging conditions in their life.

 It would also be helpful to include retrospective variables relating to the subjects perceived parenting history which has been shown in several studies to substantially predict LOC.

I have read this submission. I believe that I have an appropriate level of expertise to confirm that it is of an acceptable scientific standard.

# Response

**Published:** 2017-11-06

**Authors:** Yasmin Iles-Caven

**Affiliations:** University of Bristol, UK

This is an important study highlighting the special influence of a theoretically and empirically psychological variable impacting on the health and well-being of human beings. Apart from the especially large sample the study addresses an important single variable relating to an appropriate development of human beings’ self-determination which has been shown in a broad range of national and international studies.

Apart from an albeit large female sample the study would have profited from a cluster-analytic approach determining different constellations of predictive variables impacting on the subjects’ well-being. Moreover, a longitudinal approach to assessing the influence of external locus of control in combination with internal locus of control would allow to draw more specific influence on the subject’s well-being and potential to cope with challenging conditions in their life.

***To use a cluster approach to the analysis is an interesting idea, and one which may be carried forward in the future. Here, however, we wanted to continue the method of analyses which had started with an analysis of the women’s background and first 5 years of life [Golding J, Iles-Caven Y, Gregory S, Nowicki S. (2017) The antecedents of women’s external locus of control:  Associations with characteristics of her parents and of early childhood. Heliyon 3: doi: 10.1016/j.heliyon.2017.e00236].  The results of the present analyses have also been compared with a set of similar analyses using data concerning men in the ALSPAC study that also completed LOC proformas [paper submitted].***

***We do agree with Dr Schneewind that an analysis concentrating on internal orientation would be a valuable addition to our future strategy.***

It would also be helpful to include retrospective variables relating to the subjects perceived parenting history which has been shown in several studies to substantially predict LOC.

***We were, alas, constrained by the variables that had been collected. These did included two retrospectively obtained quality of maternal care scores. – that concerned with over-protectiveness did not enter the model, but that on the quality of care itself did.***

# Peer review: Referee response for version 1

**DOI:** 10.21956/wellcomeopenres.13038.r25817

**Published:** 2017-09-25

**Authors:** Aleksandra Kostic

**Affiliations:** 1 Faculty of Philosophy, Study Group for Psychology, University of Niš, Niš, Serbia

First, I wish to compliment the authors' initiative and I think their work will contribute to the literature in the area of ELOC. However, I do have some reservations and will like authors to substantially address.

1. The manuscript will greatly improve if a theoretical framework and a strong literature review section are inserted. This section should speak to the variables on which the analysis is based. The authors have asserted these variables into the study rather than systematically argue via the literature, their importance. 
2. The methodology section needs to systematically describe variables, providing information on number of items used, range, mean, SD, median etc.
3. Results section has not reported description of variables. This can be done in a table which point # 2 above can refer to. Also, skewness, kurtosis, linearity etc. need to be reported.
4. What percentage of data is missing for each variable? Report Little MCAR statistics. How did authors deal with missing data and outliers?
5. The discussion section is really an elaborated repeat of the result section. If point #1 above is address, this will lay the foundation for having an engaging discussion.

 

 I make these comments to encourage the authors to push the manuscript a few notches higher. The manuscript has very important information which can be more systematically presented. I encourage being guided by the APA Publication Manual.

I have read this submission. I believe that I have an appropriate level of expertise to confirm that it is of an acceptable scientific standard, however I have significant reservations, as outlined above.

# Response

**Published:** 2017-11-06

**Authors:** Yasmin Iles-Caven

**Affiliations:** University of Bristol, UK

First, I wish to compliment the authors' initiative and I think their work will contribute to the literature in the area of ELOC. However, I do have some reservations and will like authors to substantially address.

 The manuscript will greatly improve if a theoretical framework and a strong literature review section are inserted. This section should speak to the variables on which the analysis is based. The authors have asserted these variables into the study rather than systematically argue via the literature, their importance. 

***We agree that an alternative strategy would have been to choose variables based on theoretical considerations. However, we felt that by doing that we may have missed important influences on the development of ELOC. We therefore used this hypothesis free exposome approach, but have noted in the discussion ways in which the results coincide with what would have been expected on theoretical grounds.***

 The methodology section needs to systematically describe variables, providing information on number of items used, range, mean, SD, median etc.

***The very nature of the Exposome approach means that a large number of variables are used.  These are all described on the study website:*www.bristol.ac.uk/alspac/researchers/data-access/data-dictionary/. *Information on the more prominent variables is described in the Supplementary File to this paper. The study used all available data for each variable (numbers are given on the web site).***

 Results section has not reported description of variables. This can be done in a table which point # 2 above can refer to. Also, skewness, kurtosis, linearity etc. need to be reported.

***See response above. In regard to description of the variables, these were mainly categorical and not continuous.***

 What percentage of data is missing for each variable? Report Little MCAR statistics. How did authors deal with missing data and outliers?

***Again the data for each variable, including the numbers with missing data, are given on the website. Because we were not convinced that the data were missing at random, we did not impute data, but used complete data only in the analyses. We give the numbers with information available in the unadjusted sections of the Supplementary Tables.***

 The discussion section is really an elaborated repeat of the result section. If point #1 above is address, this will lay the foundation for having an engaging discussion.

***We are grateful to Dr. Kostic – and have added a section to the Discussion to put more depth into the Discussion, as well as to tie our results to a theoretical background.***

 I make these comments to encourage the authors to push the manuscript a few notches higher. The manuscript has very important information which can be more systematically presented. I encourage being guided by the APA Publication Manual.
